# Review of "Reconstructing Evolution: New mathematical and computational advances" edited by Olivier Gascuel and Mike Steel

**DOI:** 10.1186/1748-7188-2-14

**Published:** 2007-11-06

**Authors:** Andreas Spillner

**Affiliations:** 1 School of Computing Sciences, University of East Anglia, Norwich, UK

## Overview

The main theme of the book is mathematical models of evolutionary processes in biology. These models have become increasingly complex in order to cope with the various aspects involved in the study of these processes and involve a wide range of concepts from different areas of mathematics and computer science. This book gives an overview of some of the key contemporary topics in the field. The book is organized in 10 chapters, each written by an expert or a group of experts on the topic addressed in that chapter. At the end of many chapters directions for future research are discussed. Each chapter comes with its own list of references. At the end of the book the reader can find an index that helps to locate quickly the context within which a particular concept appears.

## Contents

The book starts with a brief introduction by the editors. They outline how the discovery of biological processes such as alternative splicing and lateral gene transfer have changed our view of evolution and how this has led to very complex models of evolutionary processes in biology. The editors emphasize that a better understanding of these processes is of great value for solving problems such as the prediction of protein function from genomic sequences or biodiversity conservation planning.

The first two chapters are grouped together under the headline *Evolution in Populations*. Chapter 1, by J. Felsenstein, first reviews the basic concept of *coalescents*, that is, trees that describe the ancestral relationship between copies of genes formed in populations. Next, to obtain a more realistic model, the effects of population growth, migration, recombination and natural selection are taken into account. Then, inference methods based on the model are reviewed and at the end of Chapter 1 the reader can find a list of available coalescent programmes. Chapter 2, by A. Rodrigo et al., is centered around the concept of *measurably evolving populations*, that is, populations for which sequence data is obtained over time and a significant accumulation of substitutions is found. The authors review methods for constructing evolutionary trees and estimating quantities such as substitution rate, population size and migration rate under these circumstances.

Chapters 3 and 4 are dedicated to *Models of Sequence Evolution*. The first part of Chapter 3, by O. Gascuel and S. Guindon, addresses the issue of *variability *of sequence evolution, and the reader can find an extensive overview of existing models that take this variability into account. In the second part of the chapter, two data sets are used to illustrate how these models can be employed to analyze sequence data. In Chapter 4, by E. S. Allman and J. A. Rhodes, the concept of *phylogenetic invariants *is reviewed. The authors provide the reader with the necessary basic terminology from algebraic geometry and then explain how such invariants can be found and how they can help to better understand models of sequence evolution. 

Chapters 5 and 6, *Tree Shape, Speciation, and Extinction*, focus on how to model diversification and assess biodiversity. In Chapter 5, by A. O. Mooers et al., several models are discussed for generating trees. The goal is to generate trees whose shape is similar to that of evolutionary trees constructed from real biological data and, thereby, to better understand the processes that lead to the patterns found in those trees. Chapter 6, by K. Hartmann and M. Steel, reviews a measure used in conservation planning called *phylogenetic diversity *(PD). After presenting some of the mathematical properties of this measure it is compared to other measures of biodiversity and issues like the time scale for planning or budgetary constraints are discussed. The chapter concludes with some statistical properties of PD and its application in the reconstruction of evolutionary trees.

Chapters 7 and 8 are devoted to problems arising in the context of the reconstruction of *Trees from Subtrees and Characters*. In Chapter 7, by M. J. Sanderson et al., the problem of *fragmentation *of the data in large scale phylogenetic analysis as addressed and strategies to deal with this problem are discussed. The problems studied in Chapter 8, by S. Grünewald and K. T. Huber, arise from the question: When does a data set support an evolutionary history that is tree-like? After formalizing this question, a collection of well known and also more recent combinatorial results that answer this question is reviewed, including characterizations of phylogenetic trees in terms of chordal graphs and closure rules.

The last two chapters, *From Trees to Networks*, are motivated by the fact that the evolutionary history of some sets of taxa is better represented by a network rather than a tree. Chapter 9, by D. Huson, first gives a short overview of which types of networks are currently used to represent biological data. Then several methods for computing such networks are reviewed. In Chapter 8, by C. Semple, the basic problem is to find a network that can explain the biological data involving a minimum number of hybridization events. A characterization of such networks as well as the connection to several related optimization problems along with their computational complexity is discussed.

## Summary

In my opinion the book has achieved the goal of the editors to give a detailed overview of key topics in molecular evolution. As with any selection of topics, especially in a field developing as rapidly as phylogenetics, there are certainly topics that have not been addressed but are (or soon turn out to be) of equal importance such as, e.g., the simultaneous construction of sequence alignments and evolutionary trees. The material that is covered in the book can serve as an entry point which leads the reader quickly to the relevant literature and also provides some help for how different approaches to a particular problem compare to one another. It is certainly not a textbook and, depending on the topic, requires a good deal of background knowledge. Depending on this background knowledge, some readers aiming to understand in depth the methods and results discussed will probably need to consult the original papers. In conclusion, the book can serve as a very good reference for researchers in the field and to some extent as a source where the reader searching for an appropriate method to analyze a particular data set can find some guidance.

